# Early Apple Bruise Detection via Discrete Hyperspectral Signatures with SHAP-Guided Feature Selection and a CNN–Transformer Model

**DOI:** 10.3390/foods15111884

**Published:** 2026-05-26

**Authors:** Ying Liu, Chen Yu, Chaoxian Liu, Zhilian Xu, Bin Xiong, Chengyu Zhang, Weiqiang Yang, Wei Tao

**Affiliations:** 1 School of Mathematics & Computer Science, Wuhan Polytechnic University, Wuhan 430023, China; ly_2027@163.com (Y.L.); mc_yuchen@whpu.edu.cn (C.Y.); 13385286894@163.com (Z.X.); toofth21@163.com (B.X.); taowei@whpu.edu.cn (W.T.); 2 School of Electrical Engineering, Southwest Jiaotong University, Chengdu 611756, China; zcy15927347998@outlook.com; 3 College of Information Engineering, Northwest A&F University, Yangling 712100, China; ywq19960313@outlook.com

**Keywords:** non-destructive inspection, waveband optimization, sparse spectral learning, deep learning, postharvest quality assessment

## Abstract

Accurate detection of early invisible apple bruises is important for post-harvest quality assessment. Although hyperspectral imaging (HSI) provides rich spectral information, its high dimensionality introduces substantial redundancy and weak-signal interference. This study proposes an integrated framework combining waveband optimization and discrete spectral modeling for efficient bruise detection. A Selection-Refined Improved Grey Wolf Optimization (SR-IGWO) algorithm was developed to select 18 bruise-sensitive wavebands from 273 channels (996–2501 nm), achieving a 93.4% reduction in spectral dimensionality. SHAP analysis was further used to interpret the selected bands in relation to biochemical responses associated with bruising. To address the mismatch between conventional CNNs and sparse discrete spectral inputs, a CNN–Transformer hybrid model (DSFormer) was designed using pointwise convolution for band embedding and a Transformer encoder to capture global dependencies. Experimental results across ten independent runs achieved a classification accuracy of 99.11% ± 0.08%, a recall of 96.04% ± 1.08%, and an F1-score of 95.95% ± 0.39% under the tested conditions. Ablation studies suggest that the proposed architecture supports effective detection under sparse spectral conditions. Although validation was limited to a single cultivar and controlled sampling, the proposed framework provides a promising preliminary exploration of reduced hyperspectral data for non-destructive fruit bruise detection.

## 1. Introduction

Apples are high-value horticultural products, yet their quality is frequently compromised by mechanical impacts during postharvest handling. Unlike visible decay, early bruises are often subsurface, involving cell wall rupture, intracellular fluid leakage, and enzymatic browning driven by polyphenol oxidase. These subtle physiological changes are initially masked by the epidermis, making them difficult to detect using conventional RGB imaging or manual inspection [1]. As early bruises may progressively expand and lead to secondary deterioration, rapid and non-destructive detection of these weak internal changes is essential for reliable fruit quality evaluation and automated grading [2,3].

Hyperspectral imaging (HSI) has emerged as an effective tool for fruit quality assessment by integrating spatial and spectral information. By capturing absorption features associated with water (O-H) and organic compounds such as sugars and phenolics, HSI enables early detection of tissue changes before visible symptoms appear [4]. However, conventional HSI systems rely on full-spectrum acquisition with hundreds of contiguous bands, resulting in substantial data redundancy and increased computational complexity. High spectral dimensionality may introduce additional noise and affect model optimization stability under complex experimental conditions [5]. Therefore, reducing spectral dimensionality while preserving discriminative information remains a key challenge for practical HSI applications in food quality assessment [6].

To reduce hyperspectral dimensionality in apple bruise detection, various feature selection and projection methods have been reported [7]. Principal Component Analysis (PCA) reduces dimensionality through linear transformation but often loses non-linear discriminative features and physical interpretability [8]. Competitive Adaptive Reweighted Sampling (CARS) selects variables based on regression coefficients but may suffer from instability under sample noise [9]. The Successive Projections Algorithm (SPA) alleviates multicollinearity; however, its search strategy is prone to local optima [10]. Attention-based visualization methods such as Grad-CAM [11] have also been introduced to interpret spectral importance, yet their selected regions may exhibit aggregation or redundancy. Although these approaches reduce dimensionality to some extent, simultaneously achieving global optimization capability, selection stability, and physical interpretability remains challenging [12]. Particularly in early invisible apple bruise detection, the combination of weak discriminative signals, blurred class boundaries, and high spectral dimensionality increases the risk of premature convergence and unstable band subsets [12]. Consequently, there is a need for a refined optimization strategy that ensures both selection stability and physical interpretability [13].

In parallel with band optimization research, various modeling strategies have been applied to hyperspectral fruit damage detection [14], including Partial Least Squares Discriminant Analysis (PLS-DA) [15], Support Vector Machines (SVM) [16], one-dimensional Convolutional Neural Networks (1D-CNN) [17], and recurrent neural networks. These methods have shown progressively improved capability in capturing complex spectral characteristics associated with bruising. More recently, studies have further explored spectral–spatial feature enhancement using 3D convolutional neural networks [18], NIR characteristic wavelength imaging combined with image segmentation [19], and ANN-based regression for bruise area prediction [20]. From a methodological perspective, existing hyperspectral bruise-detection studies generally follow two main technical paradigms. The first directly applies deep learning models to full-spectrum hyperspectral data, which often provides strong representation capability but suffers from substantial spectral redundancy, increased computational burden [21], and limited deployment efficiency. The second combines waveband-selection strategies with traditional machine learning classifiers to improve efficiency; however, such approaches may exhibit limited capability for modeling nonlinear interactions and global dependencies [22] among sparse discrete spectral features. In addition, most existing methods either treat the detection process as a black box without interpretability or rely on conventional pipelines that do not explicitly reveal which spectral bands contribute most strongly to the final decision. More importantly, practical waveband optimization often produces sparse and non-contiguous spectral subsets rather than continuous spectral sequences. Under such conditions, conventional convolutional or recurrent architectures that implicitly assume local spectral continuity may become structurally mismatched to the actual input characteristics, potentially weakening discriminative feature representation. Furthermore, early bruise samples usually exhibit extremely subtle spectral perturbations, resulting in many hard-to-classify instances. Traditional loss functions are often dominated by easy healthy samples, suppressing the learning of weak bruise-related signals and reducing recall performance [23,24]. Therefore, under the discrete-input framework, developing a classification model that balances global collaborative modeling with enhanced learning for hard-to-classify samples has become a critical issue.

Overall, transitioning from continuous spectra to discrete key bands is essential. However, this may introduce challenges for traditional deep learning models: 1D-CNNs and RNNs rely, to a certain extent, on local spectral continuity—an assumption that may not hold when inputs are non-contiguous [25,26]. Furthermore, the inherent “hard-to-classify” nature of weak browning signals and the challenge of capturing subtle spectral features often lead to low recall in standard models. There is a need for a framework that can both interpret discrete spectral interactions and enhance weak signal detection [27]. To address these issues, this study proposes an integrated hyperspectral measurement and modeling framework for early apple bruise detection [28,29]. The framework aims to simplify hyperspectral acquisition, reduce system burden, and improve optimization stability under discrete key band conditions. The main contributions are summarized as follows:

(1) Unlike conventional full-spectrum hyperspectral learning frameworks, a sparse waveband optimization strategy integrating SR-IGWO and SHAP analysis was introduced to reduce spectral redundancy while preserving representative bruise-sensitive spectral information and improving feature interpretability.

(2) Different from traditional classifiers operating on selected wavebands, a structurally matched CNN–Transformer framework was designed for sparse and non-contiguous spectral by combining pointwise spectral embedding with global dependency modeling, thereby improving weak bruise detection under reduced spectral dimensionality.

(3) A visualization-based detection strategy was established to enable spatial localization of bruise regions, supporting intuitive interpretation of early damage distribution.

## 2. Materials and Methods

### 2.1. Overall Framework

**Figure 1:**
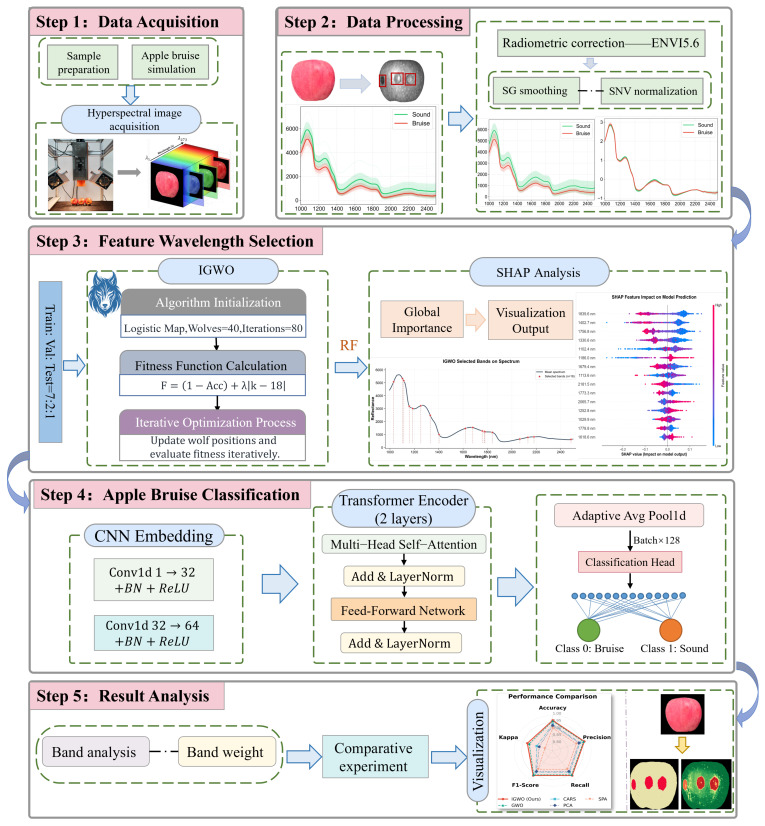
Overview of the proposed framework for early apple bruise detection.

As illustrated in Figure 1, a structured and synergistic framework was proposed for the non-destructive detection of early apple bruises. The workflow consists of five sequential stages. First, hyperspectral images were acquired following sample preparation and controlled bruise simulation, and regions of interest (ROIs) were extracted to obtain representative spectral signals. Second, raw spectra were preprocessed through radiometric correction, Savitzky–Golay (SG) smoothing, and Standard Normal Variate (SNV) normalization to reduce noise, baseline drift, and scattering effects. Third, the Selection-Refined Improved Grey Wolf Optimization (SR-IGWO) algorithm was employed to identify a compact subset of informative wavebands, significantly reducing spectral redundancy. Subsequently, SHAP analysis was introduced to interpret the contribution and physical relevance of selected bands. Finally, the optimized features were input into the DSFormer model for classification, where Focal Loss was applied to enhance sensitivity and increase attention to hard-to-classify bruise samples. The framework concludes with a comprehensive evaluation, including quantitative metrics, comparative experiments, and visualization-based spatial analysis of bruise regions.

### 2.2. Sample Preparation and Bruise Induction

One hundred Fuji apples were harvested from a commercial orchard in Weinan, Shaanxi, China. Fuji apples were selected in this study because their relatively dense parenchymal tissue and thicker epidermis may affect early bruise manifestation compared with thinner-skinned cultivars such as Gala or Golden Delicious. To ensure sample consistency, only apples with uniform commercial maturity were used, with an average firmness of approximately 75 N, a soluble solids content of approximately 14.0° Brix, equatorial diameter of 80 ± 5 mm, and average weight of 220 ± 20 g. All samples were manually harvested and transported under padded conditions to minimize unintended mechanical damage prior to experimentation. Before the controlled impact tests, each apple was visually inspected under uniform halogen illumination to exclude samples with visible defects, scratches, soft spots, or apparent pre-existing bruises. Because this study focuses on early invisible bruising, microscopic or early subsurface damage cannot be completely ruled out through visual inspection alone. Therefore, the pre-screening procedure was primarily intended to ensure the absence of observable surface defects and to minimize uncontrolled mechanical interference before standardized bruise induction. To stabilize metabolic rates and minimize physiological variability, all samples were equilibrated at 20 °C and 75% relative humidity for 24 h prior to experimentation [30].

**Figure 2:**
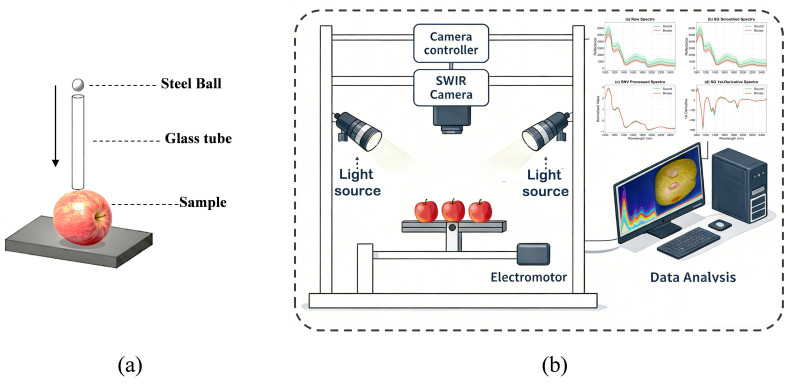
Schematic diagram of apple bruise sample data acquisition. (**a**) Apple bruise simulation device. (**b**) Hyperspectral acquisition device.

Early bruising was simulated through controlled mechanical impacts. As shown in Figure 2a, we employed a controlled drop test using a 2.5 cm diameter solid stainless-steel ball (density: 7.93 g/cm^3^, mass: 64.9 g) from heights of 10 cm, 15 cm, and 25 cm [31]. Based on the potential energy equation *E* = *mgh*, these drop heights generated mechanical impact energies of approximately 0.064 J, 0.095 J, and 0.159 J, respectively. These specific mild-impact energies were deliberately selected to induce highly localized, weak-signal physiological browning while maintaining visually intact epidermal surfaces. A total of 75 apples were randomly assigned to the bruise group, with 25 apples subjected to each impact level (10, 15, and 25 cm), while the remaining 25 apples were retained as the control group without mechanical impact. To isolate the impact of browning from microbial interference, both the sphere and the fruit surface were disinfected with 75% ethanol. Impacts were localized at the equatorial region to concentrate mechanical energy within the parenchyma while maintaining epidermal integrity.

Following impact, apples were stored for 1–4 h—a critical window where cellular disruption and enzymatic browning occur without visible surface deterioration—thereby capturing the “early invisible” stage of damage [32]. To ensure reliable ground-truth labeling of early invisible bruises, the impact locations were first recorded during the mechanical simulation. Immediately after hyperspectral acquisition, suspected regions were preliminarily identified based on the known impact coordinates and slight tactile differences. The samples were then stored for an additional 10 h to allow bruise development, after which the corresponding regions were visually inspected to confirm subepidermal browning. Only regions with consistent positional correspondence and visible browning were labeled as bruised, providing a consistent reference for ROI extraction and subsequent analysis.

### 2.3. Hyperspectral Data Acquisition

Spectral data were acquired using a SPECIM SWIR hyperspectral system (SPECIM, Oulu, Finland) operating in the 996–2501 nm range with 273 bands at a approximately 5 nm resolution (Figure 2b). The imaging sensor had a spatial resolution of 384 pixels and included a semiconductor cooling module to reduce thermal noise [33]. Samples were placed on a LabScanner 100 × 50 motorized translation stage (Specim, Spectral Imaging Ltd., Oulu, Finland), and illuminated by 14 symmetrically arranged 12 V/100 W halogen lamps. The lamp group formed a 45° angle with the sample plane to ensure uniform illumination in the range of 350–2500 nm. The acquisition parameters were set as follows: integration time of 80 ms, lens-to-sample surface distance of 30 cm, and translation stage moving speed of 2 mm/s to avoid motion blur [34]. To eliminate system noise and environmental interference, standard white and dark reference board correction was performed before data acquisition [35]. The full white reference board image ($I_{white}$) and the dark field image with the lens completely covered ($I_{dark}$) were collected separately, and the reflectance correction of the original hyperspectral image ($I_{origin}$) was conducted according to Equation (1):

(1) $$\;I_{corrected}=\frac{I_{origin}-I_{dark}}{I_{white}-I_{dark}}$$

where $I_{corrected}$ denotes the calibrated reflectance image. The corrected data were directly used for subsequent ROI extraction and spectral analysis to ensure data quality and reliability. In addition, reproducibility assessment was conducted using 10 randomly selected apples. Each sample was scanned three times under identical acquisition settings. To simulate practical measurement variability, full repositioning was performed between scans, which involved manually removing the sample from and re-placing it onto the translation stage [36]. For each selected waveband, the coefficient of variation (CV) was calculated as the ratio of the standard deviation to the mean reflectance across the three repeated scans at the corresponding region of each apple [37]. The average CV across all key wavebands was below 3%, indicating relatively low variability and high measurement repeatability under repositioning conditions. This procedure reflects both instrumental stability and measurement consistency under sample repositioning conditions, supporting the measurement consistency of the hyperspectral acquisition system.

### 2.4. ROI Extraction for Apple Bruise Regions

**Figure 3:**
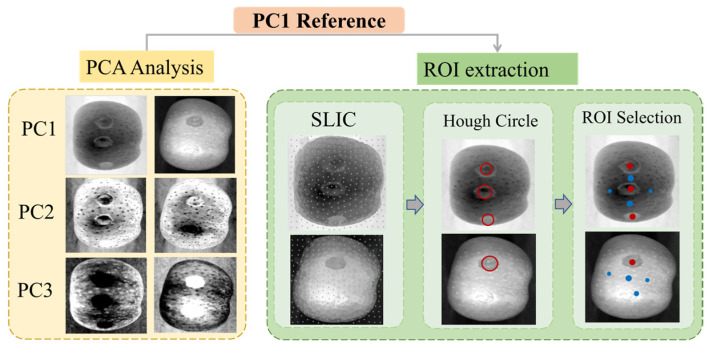
ROI extraction process for apple bruises.

Given the difficulty of visually identifying early bruises, a semi-automated ROI extraction strategy was adopted to improve efficiency and consistency. To mitigate spherical effects and illumination gradients, a multi-step procedure integrating hyperspectral processing and image segmentation was implemented (Figure 3). First, PCA was applied to the hyperspectral data, and the first principal component (PC1) was selected as the reference image due to its superior contrast and noise suppression in representing tissue morphology [38]. Second, the Simple Linear Iterative Clustering (SLIC) algorithm [39] (K = 800, compactness = 10) was used to generate superpixels, facilitating boundary refinement. The apple contour was then detected using the Hough Circle Transform, and ROIs were manually annotated in ENVI 5.6 based on superpixel boundaries and recorded impact locations [40].

Each ROI contained approximately 200–400 pixels and was restricted to the equatorial region to ensure uniform illumination conditions [41]. All annotations were performed by a trained researcher following a standardized protocol and were cross-validated against post-storage browning verification (Section 2.2) and recorded impact coordinates. Each apple contained approximately 6–8 bruised regions induced by controlled impacts (multiple ROIs can be marked within each individual damaged area). Across the dataset, more than 5700 bruised ROIs were annotated, along with corresponding healthy ROIs from adjacent intact tissues. To mitigate pixel-level noise, the reflectance spectra of all pixels within each individual ROI were averaged to extract an object-level mean spectrum, which constitutes a single object-level spectral sample. Each sample consisted of 273 spectral bands with a binary label (0: bruised, 1: healthy). In total, the dataset comprised over 11,000 samples (approximately 5700 bruised and 6000 healthy). The object-level average spectra derived from these ROIs provided a stable basis for subsequent model development under the current dataset.

### 2.5. Wavelength Optimization via SR-IGWO

Hyperspectral datasets are characterized by high dimensionality, severe redundancy, and strong multicollinearity among adjacent channels [42]. Directly inputting all 273 bands into deep learning models increases computational complexity and introduces redundant noise, which often degrades model generalization [39]. To address this, a Selection-Refined Improved Grey Wolf Optimization (SR-IGWO) algorithm was developed. This framework identifies a sparse subset of discriminative wavebands by embedding a Random Forest (RF) classifier [43] as a fitness evaluator. Furthermore, SHAP analysis [44] was integrated to validate the physical relevance of the selected bands, ensuring the model’s decision-making aligns with the biochemical fingerprints of apple tissue. The SR-IGWO systematically purifies the signal via Savitzky–Golay (SG) smoothing and employs a synergistic search strategy to navigate the high-dimensional spectral space. The detailed execution is summarized in Algorithm 1.
**Algorithm 1.** SR-IGWO for Waveband Selection**Input:** Preprocessed data $D_{data}$; label Y; target dimension *K*; number of wolves *W*; max iterations $T_{max}$; penalty λ
**Output:** Optimal discrete waveband subset $S_{best}$
1.        Initialize population $X_{i}\in {\left[0,L-1\right]}^{K}$(*i* = 1, …, *W*) using logistic chaotic mapping. (Equation (2)) (Total number of original wavebands *L* (*L* = 273))2.        Initialize leader wolves: $X_{\alpha }$, $X_{\beta }$, and $X_{\delta }$, and their fitness $F_{\alpha }=F_{\beta }=F_{\delta }=+\infty$3.        while *t* $\leq T_{max}$ do4.        for each wolf *i* from 1 to *W*:5.        decode to valid discrete subset: $S_{i}\leftarrow Unique(Round\left(Clip\left(X_{i},0,L-1\right)\right))$
6.        calculate internal RF classification accuracy based on $S_{i}$, $D_{data}$, and Y.7.        evaluate hybrid fitness (Equation (4))8.        update leaders $X_{\alpha }$, $X_{\beta }$, $X_{\delta }$ according to the best three $F_{i}$ values9.        end for10.      update the non-linear convergence factor a(t). (Equation (3))11.      for each wolf *i* from 1 to *W*:12.      for k∈{α,β,δ} do 13.      calculate step vectors with random coefficient vectors $A_{k}$
14.      (controlled by a(t)) and $C_{k}$: $V_{k}\leftarrow X_{k}-A_{k}⊙{|C}_{k}⊙X_{k}-X_{i}|$15.      end for16.      update continuous position: $X_{i}\leftarrow \frac{V_{\alpha }+V_{\beta }+V_{\delta }}{3}$17.      End for18.      *t* = *t* + 1.19.      end while20.      return $S_{best}=Unique(Round\left(Clip\left(X_{\alpha },0,L-1\right)\right))$


#### 2.5.1. Strategic Improvements for Spectral Search

**Figure 4:**
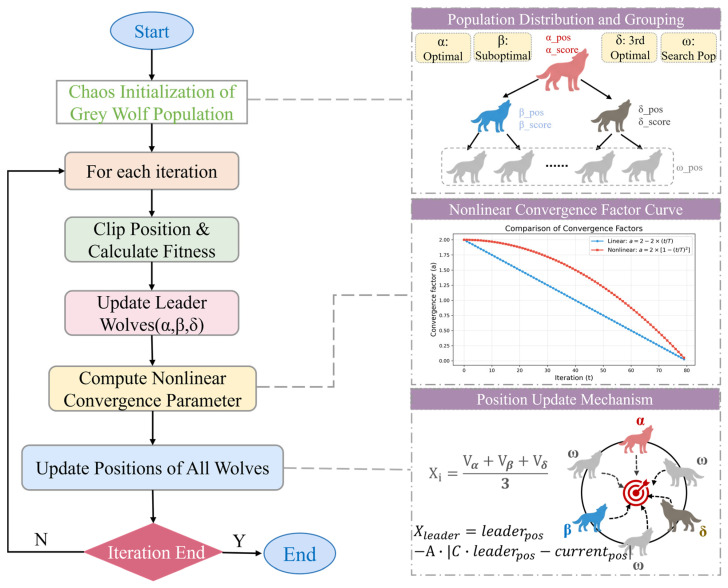
Flowchart of the improved grey wolf optimization (IGWO) algorithm.

The Grey Wolf Optimizer (GWO) [2] is a swarm intelligence algorithm inspired by the hierarchical hunting behavior of grey wolves. In the solution space, the grey wolf population is divided into four grades: α (optimal solution), β (suboptimal solution), δ (third optimal solution), and ω (remaining candidate solutions). During the standard hunting process, the continuous position of the i-th wolf ($X_{i}$) is updated by averaging the guided step vectors ($V_{k}$) from the three leaders ($X_{k}$,k∈{α,β,δ}). These step vectors are mathematically defined as $V_{k}=X_{k}-A_{k}⊙|C_{k}⊙X_{k}-X_{i}|$, where $A_{k}$ and $C_{k}$ are independent random coefficient vectors generated for each leader, dynamically controlled by a convergence factor. For the waveband selection task, the position vector of each grey wolf represents a candidate waveband subset. To enhance search robustness in the complex spectral landscape of early bruises, three critical modifications were introduced (Figure 4).

(1) Chaotic Initialization Based on Logistic Mapping

To improve population diversity and avoid premature convergence, the traditional random initialization strategy was abandoned in this study [45]. Logistic chaotic mapping was adopted to generate the initial population positions, and the ergodicity and regularity of chaotic variables were utilized to ensure that the initial solutions were uniformly distributed in the waveband index space. The iterative equation is given as follows:

(2) $$\;v_{k+1}=\mu \cdot v_{k}(1-v_{k})$$

where $v_{k}$ denotes the chaotic variable at the *k*-th generation, and μ is the control parameter (set to 4.0 in this study). Under this condition, the sequence exhibits chaotic behavior, promoting uniform distribution of initial solutions within the waveband index range [0, 272].

(2) Nonlinear Convergence Factor

In the original GWO, the convergence factor *a* decreases linearly. To balance the global exploration and local exploitation capabilities of the algorithm, a nonlinear attenuation curve based on a quadratic function was designed:

(3) $$\;a(t)=2(1-({\frac{t}{T_{max}})}^{2})$$

where t is the current number of iterations, and $T_{max}$ is the maximum number of iterations (set to 80 in this study). In the early stage of iteration, the value of a(t) decreases slowly, prompting individuals to conduct extensive searches in the entire spectral range; in the later stage of iteration, it decreases rapidly, guiding individuals to perform local precise exploitation. The improved algorithm can obtain strong global search capability in the early stage of the search and achieve fine local search thereafter.

(3) Wavelength Coding Mapping

The position of grey wolves is a continuous value and needs to be mapped to discrete waveband indices. Aiming at the discrete characteristics of hyperspectral waveband selection, a coordinate rounding mapping strategy was adopted to establish the connection between the continuous solution space and physical waveband indices. The dimension of a grey wolf individual was set to the target number of bands *K*, and the position vector of individual *i* was expressed as a multi-dimensional vector $X_{i}\in R^{K}$. The continuous position vector was projected into the boundary [0, *L* − 1], and rounded to an integer. After the mapping was completed, the generated index sequence was deduplicated to extract a unique discrete waveband subset $S_{i}=Unique(Round\left(X_{i}\right))$ to ensure the validity of the feature subset.

#### 2.5.2. RF-Driven Fitness Function

The fitness function is used to evaluate the classification performance of waveband subsets. In this model, to accurately evaluate the discriminability of feature subsets for nonlinear spectral data, the RF classifier was embedded in the optimization loop as an internal evaluator. During each iteration, the candidate waveband subset was evaluated using an internal stratified 75/25 train-validation split within the optimization data pool to calculate the classification accuracy. The stratification ensures a consistent class distribution across splits, mitigating the risk of evaluation bias caused by random sampling fluctuations, thereby maintaining the representative, balanced nature of the original dataset. A hybrid fitness function $F_{i}$ was constructed as follows:

(4) $$\;F_{i}=\left(1-Accuracy\right)+\lambda \left|\left|S_{i}\right|-K|\right|$$

where *Accuracy* is the classification accuracy of the random forest on the internal validation split; $\left|S_{i}\right|$ is the number of unique wavebands in the current subset $S_{i}$; *K* is the target number of wavebands (set to 18 in this study); and λ is the penalty coefficient (set to 0.0001 in this study). This specific penalty term is introduced as a soft constraint: the small magnitude of λ ensures that the algorithm gently guides the swarm towards the target dimensionality (*K*) without overwhelming the primary objective of minimizing the classification error. This formulation simultaneously considers classification performance and subset compactness, encouraging the selection of highly discriminative yet low-dimensional waveband combinations [46].

#### 2.5.3. SHAP Interpretability Analysis

To bridge the gap between machine learning and food bioscience, SHAP analysis was performed on the optimized RF model. SHAP is a model interpretation method based on cooperative game theory, whose core idea is to convert the output of complex black-box models into interpretable additive feature contribution values by quantifying the marginal contribution of each feature to the model prediction results. For a dataset containing *M* features, SHAP evaluates the importance of feature *i* by calculating the expected value of its marginal contribution across all possible feature subsets *S*, and the calculation formula is as follows:

(5) $$\phi_{i}=\sum_{S\subseteq \{x_{1},\ldots ,x_{M}\}\{i\}}\frac{\left|S\right|!\left(M-\left|S\right|-1\right)!}{M!}[\upsilon \left(S\cup \left\{i\right\}\right)-\upsilon \left(S\right)]$$

where $\phi_{i}$ denotes the SHAP value of waveband *i*, reflecting the average marginal contribution of this feature to the model prediction results; *M* is the total number of features; $\left|S\right|$ is the size of subset *S*; $\upsilon \left(S\right)$ is the model prediction value corresponding to the feature subset *S*; and $\upsilon \left(S\cup \left\{i\right\}\right)$ is the model prediction value after adding feature *i*.

It should be noted that the SHAP analysis was applied to the RF classifier that served as the internal fitness evaluator during the SR-IGWO selection process, rather than directly interpreting the DSFormer model. The subsequent association between selected wavebands and biochemical characteristics (e.g., water absorption or cell wall structure) [47] is therefore considered as a plausible interpretation based on known spectral assignments, rather than experimentally validated causal evidence. Therefore, the SHAP results should be interpreted as supportive evidence for feature relevance [48]. This analysis provides a transparent reference for understanding the contribution of selected bands and supports the rationality of the band optimization results.

### 2.6. DSFormer Architecture for Bruise Classification

**Figure 5:**
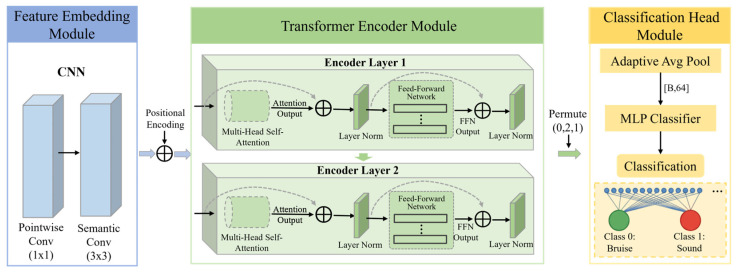
Architecture of the proposed DSFormer network.

The 18 wavebands identified by SR-IGWO are discretely distributed, violating the implicit assumption of spectral continuity required by conventional CNNs [49]. To address the structural mismatch caused by non-contiguous inputs, we developed DSFormer, a hybrid network specifically engineered for discrete spectral morphologies. Unlike sliding-window convolutions that introduce artificial local coupling, DSFormer employs pointwise embedding to preserve the independence of each waveband, followed by a Transformer encoder [50] to model global cross-band dependencies. As illustrated in Figure 5, the architecture comprises three functional modules: hierarchical feature embedding, a Transformer-based global encoder, and a classification head.

#### 2.6.1. Hierarchical Feature Embedding Module

To avoid the misinterpretation of local continuous patterns, a discrete-tailored embedding strategy was implemented. First, a pointwise convolution layer (kernel size = 1, input channels = 1, output channels = 32) independently projects the intensity of each waveband into a higher-dimensional latent space:

(6) $$\;F_{embed}=\sigma (BN\left({Conv}_{1\times 1}\left(X\right)\right))$$

where $X\in R^{B\times 1\times K}$ denotes the input discrete spectral matrix, and $F_{embed}$ represents the embedded high-dimensional representation. This operation performs channel-wise linear transformation without aggregating neighboring bands, thereby preserving the independence of physically discontinuous wavebands. To mitigate overfitting and stabilize training, Batch Normalization (BN) and a Dropout layer (rate = 0.2) are incorporated. Subsequently, a lightweight 1D convolution layer (kernel size = 3, padding = 1, output channels = 64) is applied within the learned semantic space to enable limited feature interaction. This design enhances representation optimization stability while avoiding strong assumptions about spectral continuity.

#### 2.6.2. Transformer Global Encoder Module

The physiological changes in bruised apples (e.g., the simultaneous shift in water-related and phenolic-related signatures) manifest as synergistic variations across discrete bands. To capture these long-range dependencies, a Transformer encoder was integrated. A learnable positional encoding *P* is added to $F_{embed}$ to retain waveband index information. The Multi-Head Self-Attention (MHSA) mechanism then projects the sequence into Query (*Q*), Key (*K*), and Value (*V*) matrices. The attention scores are calculated as:

(7) $$\;Attention\left(Q,K,V\right)=Softmax\left(\frac{{QK}^{T}}{\sqrt{d_{k}}}\right)V$$

where $\sqrt{d_{k}}$ is a scaling factor used to ensure numerical stability during training. Based on empirical tuning, the hidden dimension ($d_{k}$) is set to 64. By stacking L = 2 Transformer encoder layers with 4 parallel attention heads ($n_{head}=4$) and a dropout rate of 0.2, the model can adaptively learn the correlation weights among wavebands. This module enables adaptive weighting of waveband interactions, supporting discrimination of bruise-related spectral combinations under sparse input conditions.

#### 2.6.3. Classification Head and Loss Function Optimization

The feature sequence encoded by the Transformer is compressed into a single contextual vector via Global Adaptive Average Pooling (GAP) to retain the overall information of spectral responses. The classifier adopts a Multi-Layer Perceptron (MLP) structure, with Dropout introduced to alleviate overfitting. Because early bruise samples exhibit subtle differences from healthy samples, Focal Loss is adopted in this study to replace the traditional cross-entropy loss function [51]. This loss function guides the model to focus more on detecting and capturing weak spectral signal features in bruise samples with low prediction confidence by reducing the weight of easy-to-classify healthy samples, and its formula is defined as follows:

(8) $$\;FL\left(p_{t}\right)=-\alpha_{t}{\left(1-p_{t}\right)}^{\gamma }log(p_{t})$$

where $p_{t}$ denotes the predicted probability for the true class. In this study, the focusing parameter is set to γ=2.0, and the weight factor for the bruise class is set to α=0.8 to explicitly prioritize the extraction of latent damage features. This configuration enhances the model’s ability to detect faint early bruises while maintaining stable convergence behavior.

### 2.7. Experiment Setting

All experiments were conducted under a unified hardware and software environment to ensure fair comparison. The platform consisted of an Intel Core i7-12700H CPU (2.30 GHz), 16 GB RAM, and an NVIDIA GPU with CUDA support. The operating system was 64-bit Windows, and all models were implemented in Python 3.9 using the PyTorch framework (CUDA 11.8).

For waveband selection, the SR-IGWO algorithm was initialized with 40 search agents and executed for a maximum of 80 iterations to identify 18 representative wavebands. The optimization process was performed exclusively on the combined training and validation datasets. A fixed random seed (seed = 42) was used to obtain the final waveband subset for subsequent SHAP analysis and model training. To assess the optimization stability of band selection, a stability analysis was conducted over 20 repeated optimization runs with different random seeds, and the selection frequency of each waveband was statistically analyzed.

For classification, the DSFormer model was trained with a batch size of 32 for 200 epochs using the AdamW optimizer (weight decay = 0.001). A cosine annealing schedule was applied to adjust the learning rate dynamically (initial learning rate = 0.001, minimum learning rate = 1 × 10^−5^). Focal Loss (γ=2.0,α=0.8) was consistently adopted across all experiments to focus on hard-to-classify samples and enhance sensitivity to weak spectral features of early bruise signals. To account for optimization variability and improve within-dataset reproducibility, all classifiers (including baseline models and DSFormer) were independently trained and evaluated over ten independent runs with different random seeds (42, 1024, 2026, 888, 999, 123, 456, 789, 2025, and 314) [52]. The reported results are presented as mean ± standard deviation. The model obtained from the representative run (seed = 42) was further used for convergence analysis and pixel-level spatial visualization to facilitate consistent qualitative analysis across experimental demonstrations.

#### 2.7.1. Dataset Partitioning

Based on the sample preparation and splitting described in Section 2.2, over 11,000 independent spectral samples, including approximately 5700 bruised samples and 6000 sound samples, were collected in this study. To improve sample independence and minimize potential data leakage, dataset partitioning was performed strictly on an individual fruit basis. All ROIs extracted from the same apple were assigned exclusively to one subset: training (70% of fruits), validation (20%), or testing (10%). This design ensures that no spectral information from the same fruit appears in multiple subsets, thereby avoiding bias caused by spatial correlation or fruit-specific characteristics [53].

To ensure that the selected bands retain representative discriminative capability within the current dataset without compromising the integrity of the unseen test set, the SR-IGWO algorithm exclusively utilizes the optimization data pool (comprising the training and validation sets) for feature selection. During the optimization process, the fitness evaluation dynamically partitions this optimization pool into an internal stratified 75/25 train-validation split to guide feature selection. This strategy ensures that the final 18 selected bands reflect the most representative biochemical changes associated with early damage across various impact levels and control samples, while strictly isolating the final test set from the feature optimization phase [54].

Following the waveband selection, the DSFormer classification model and all comparative variants were trained and evaluated. To mitigate spatial redundancy between adjacent pixels and accelerate model convergence, a 15% random stratified sample was extracted from the designated training batch. To ensure a fair and consistent relative comparison across all evaluated models, a unified training and evaluation protocol was adopted. The 20% validation batch was utilized for internal loss monitoring and parameter tuning. The independent test batch (comprising the remaining 10% of the apples) was employed as the evaluation set to characterize performance and guide the model selection protocol consistently for all compared methods. All reported quantitative performance metrics (e.g., accuracy, F1-score) as well as the pixel-level full-surface predictions for image reconstruction were derived from this physically independent evaluation batch. Achieving high classification accuracy on this fruit-wise isolated test set suggests that the network has learned representative spectral patterns associated with early-stage damage within the current dataset, rather than merely memorizing local spatial correlations from the training fruits.

#### 2.7.2. Quantitative Evaluation Metrics

To comprehensively evaluate classification performance, multiple metrics [55,56,57,58] were adopted, including *Accuracy*, *Precision*, *Recall*, *F*1-*Score*, and Cohen’s *Kappa* coefficient.

*Accuracy* measures the overall classification correctness of the model, defined as:

(9) $$\;Accuracy=\frac{TP+TN}{TP+TN+FP+FN}$$

where TP,TN,FP, and FN represent the number of true positives, true negatives, false positives, and false negatives, respectively.

*Precision* reflects the proportion of actually bruised samples among those predicted as “bruised” by the model, with the calculation formula:

(10) $$\;Precision=\frac{TP}{TP+FP}$$

*Recall* quantifies the proportion of successfully detected bruised samples out of all actual bruised samples, calculated as:

(11) $$\;Recall=\frac{TP}{TP+FN}$$

*F*1-*Score* is the harmonic mean of *Precision* and *Recall*, used to comprehensively evaluate the model’s balanced performance between precision and recall. Its specific calculation formula is:

(12) $$\;F1-Score=2\times \frac{Precision\times Recall}{Precision+Recall}$$

To further account for potential evaluation bias and ensure classification robustness, Cohen’s *Kappa* coefficient is introduced to correct and evaluate the model performance. The *Kappa* coefficient measures the consistency between predicted results and true labels, with the calculation formula:

(13) $$\;Kappa=\frac{P_{0}-P_{e}}{{1-P}_{e}}$$

where $P_{0}$ denotes the observed accuracy and $P_{e}$ is the expected value of random consistency. A *Kappa* value closer to 1 indicates higher reliability of classification outcomes.

To complement qualitative visualization results of apple bruise, two standard spatial metrics—Intersection over Union (*IoU*) and Dice Coefficient—were employed to quantify the agreement between predicted regions (*P*) and ground truth masks (*G*). IoU evaluates the overlap ratio between prediction and reference regions, while *Dice* reflects the harmonic mean of spatial precision and recall [54]:

(14) $$\;IoU=\frac{\left|P\cap G\right|}{\left|P\cup G\right|}$$

(15) $$\;Dice=\frac{2|P\cap G|}{\left|P|+|G\right|}$$

where |P∩G| represents the number of correctly predicted bruise pixels, and |P| and |G| represent the total number of bruise pixels in the prediction and ground truth, respectively. Both metrics range from 0 to 1, with values closer to 1 indicating superior spatial boundary reconstruction.

## 3. Experimental Results and Analysis

### 3.1. Spectral Characterization and Preprocessing Analysis

**Figure 6:**
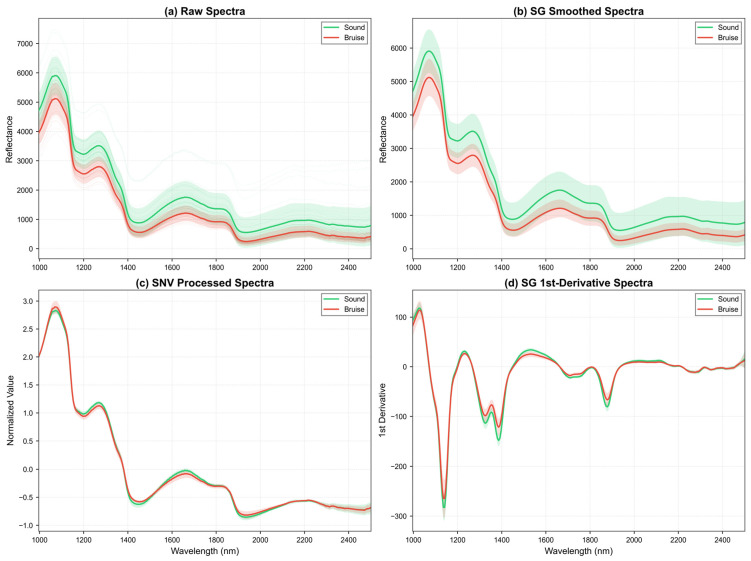
Comparison of spectral characteristics between bruised and sound apple tissues under different preprocessing strategies. (**a**) Raw spectra; (**b**) SG smoothing; (**c**) SNV; (**d**) SG first derivative.

As a preliminary overview of the dataset, to elucidate the optical response of early apple bruises in the short-wave infrared (SWIR, 996–2501 nm) range, a comparative analysis between bruised and sound tissues was conducted. As illustrated in Figure 6a, both categories exhibit similar spectral topologies, characterized by prominent absorption troughs near 1450 nm and 1940 nm, which correspond to the first overtone and the combination band of O-H stretching and bending vibrations in liquid water, respectively. While bruised tissues generally exhibit lower reflectance compared to sound tissues—likely due to increased light absorption by enzymatic browning products and altered light scattering in ruptured parenchyma cells—significant spectral overlap persists in the raw reflectance space, resulting in relatively low global quantitative separability at this initial stage. This overlap is exacerbated by the “spherical effect” of the fruit geometry and inherent biological variability among individual samples.

To enhance the discriminative signals, several preprocessing strategies were evaluated (Figure 6b–d). SG smoothing effectively suppressed high-frequency instrumental noise [59], while Standard Normal Variate (SNV) transformation mitigated the scale variations induced by surface curvature [60]. SG first-order derivative [61] was further applied to resolve overlapping peaks and eliminate baseline drift. However, despite these refinements, no distinct global separation between the two classes was observed in the full-spectrum domain. The subtle nature of early bruise-induced spectral variations, when compared to background biological noise, suggests that conventional full-spectrum preprocessing alone is insufficient. Consequently, directly computing global separability metrics on the full-spectrum data may lead to low and potentially misleading results due to substantial spectral overlap. This observation highlights the necessity of targeted waveband optimization and advanced feature modeling to extract latent diagnostic signatures of early bruising. Therefore, instead of relying on full-spectrum separability analysis, a more meaningful quantitative evaluation is conducted on the optimized feature subset using SHAP interpretation together with local statistical separability analysis in the subsequent section (Section 3.2.1).

### 3.2. Interpretable Waveband Selection and Feature Analysis

#### 3.2.1. Quantitative Contribution Analysis via SHAP

**Figure 7:**
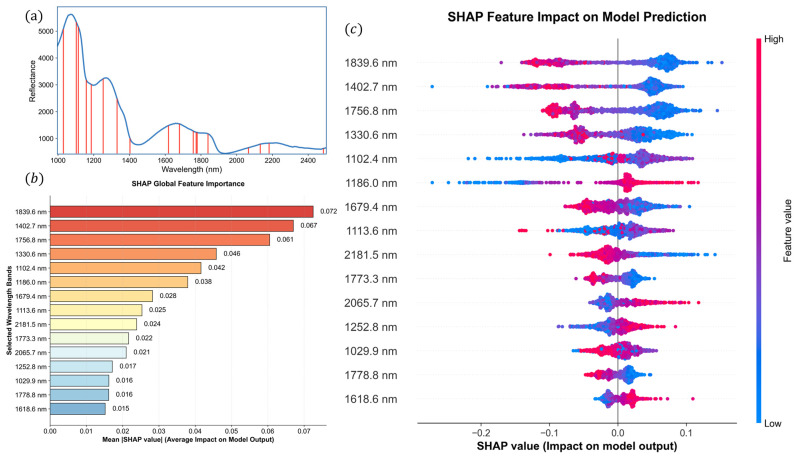
SR-IGWO waveband selection results and SHAP interpretability analysis. (**a**) Distribution of selected feature wavebands; (**b**) SHAP global importance; (**c**) SHAP beeswarm plot.

The SR-IGWO algorithm streamlined the original 273 variables into 18 key wavebands, achieving a 93.4% reduction in spectral dimensionality. The specific distribution of these selected feature wavebands across the full continuous spectrum is intuitively mapped in Figure 7a. To interpret the model decision process, SHAP analysis was performed to quantify the marginal contribution of each band (Figure 7). The global importance ranking (Figure 7b) reveals a long-tailed distribution, where the 1839.6 nm, 1402.7 nm, and 1756.8 nm bands exhibit the highest feature importance. The SHAP beeswarm plot (Figure 7c) further elucidates the directional influence of these features: for the primary band at 1839.6 nm, lower reflectance values (represented by blue dots) consistently correspond to positive SHAP values. This indicates that reflectance attenuation at this specific wavelength is a consistent indicator of bruising across most samples.

#### 3.2.2. Local Separability Analysis of Selected Bands

To further evaluate whether the SR-IGWO-selected wavebands exhibited local discriminative capability beyond model-driven feature importance, a local separability analysis was conducted on all 18 selected wavebands. Considering that hyperspectral reflectance values may not strictly satisfy the normality assumption, a non-parametric statistical framework was adopted. Specifically, the Mann–Whitney U test was used to evaluate inter-class differences between bruised and sound tissues for each selected waveband. Holm-Bonferroni correction was further applied to control the family-wise error rate across multiple waveband comparisons. In addition, Cliff’s delta was calculated as a non-parametric effect size to quantify the practical magnitude of local class separability [62,63].

**Figure 8:**
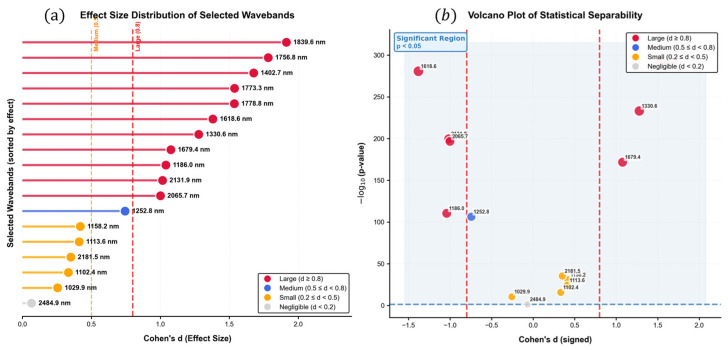
Local statistical separability analysis of the selected wavebands. (**a**) Absolute Cliff’s delta effect sizes ranked in descending order. Dashed lines indicate large ($\left|\delta \right|=0.474$) and medium ($\left|\delta \right|=0.33$) effect-size thresholds. (**b**) Volcano plot showing the relationship between signed Cliff’s delta and ${-log}_{10}$ Holm-adjusted *p*-value. The shaded region indicates adjusted *p* < 0.05.

As shown in Figure 8, 15 out of the 18 selected wavebands showed significant inter-class differences before correction (*p* < 0.05), and 14 wavebands remained significant after Holm-Bonferroni correction (adjusted *p* < 0.05). The average absolute Cliff’s delta across all selected wavebands was 0.4584, with a median value of 0.5682, indicating an overall moderate-to-large local separability pattern. In particular, 10 wavebands exhibited large effect sizes (|δ|≥0.474), with the strongest separability observed at 1839.6 nm ($\left|\delta \right|=0.8215$), 1756.8 nm ($\left|\delta \right|=0.8025$), and 1402.7 nm ($\left|\delta \right|=0.7829$). These highly separable wavebands are consistent with the SHAP-based importance analysis, suggesting agreement between model-derived feature relevance and intrinsic spectral discriminability. It should also be noted that the SR-IGWO process aims to maximize the complementary discriminative capability of the waveband subset rather than selecting wavebands solely according to individual univariate separability. Therefore, several selected wavebands with small or negligible individual effect sizes may still contribute complementary information within the multivariate feature combination. Overall, the non-parametric local separability analysis provides statistical support that the selected discrete wavebands retain meaningful discriminative information within the current dataset while maintaining consistency with the statistical framework used in the subsequent analysis.

#### 3.2.3. Selection Stability Analysis

**Figure 9:**
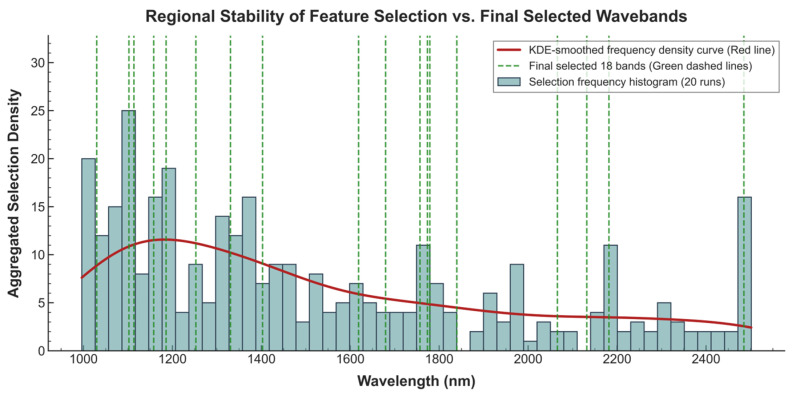
Regional stability analysis of the SR-IGWO feature selection.

To explicitly assess the optimization stability of the selected subset and address the inherent stochasticity of heuristic optimization, a stability analysis was conducted across 20 repeated optimization runs using varying random seeds [64]. Although exact discrete waveband indices exhibited slight stochastic variations across trials—a common phenomenon due to the strong multicollinearity among contiguous hyperspectral channels—the core spectral regions demonstrated consistent recurrence. As illustrated in the selection density distribution (Figure 9), the aggregated selection frequencies form distinct regional peaks, indicating that the algorithm consistently prioritizes specific spectral intervals. Specifically, high-density selection zones are concentrated in ranges such as 1100–1200 nm, 1300–1450 nm, 1700–1850 nm, and 2100–2500 nm, with average selection frequencies exceeding 85% for these core regions. Crucially, the final 18 discrete wavebands utilized for the classification model (represented by vertical dashed lines) align closely with these prominent density peaks. This repeated-run regional consistency suggests that the SR-IGWO framework systematically targets physically meaningful spectral signatures associated with tissue damage, rather than converging on random noise artifacts.

#### 3.2.4. Biochemical Implications of Selected Wavebands

To examine whether the selected wavebands correspond to known spectral absorption characteristics, the 18 selected bands were compared with reported molecular vibration regions of major chemical components in apple tissues [65]. As shown in Table 1, previous studies have shown that overtone and combination absorptions in the SWIR region are associated with O–H and C–H bond vibrations, which are related to water content, soluble carbohydrates, and structural polysaccharides [66].

The selected wavebands are broadly consistent with these established physiological principles, particularly the highly contributing features identified in the SHAP analysis. The most prominent primary band at 1839.6 nm is located within the combination absorption region of O–H and C–O stretching modes, which is strongly associated with bound water and cellulose structure. Mechanical impact may induce structural disruption of the cellular matrix, potentially leading to distinct reflectance attenuation at this specific wavelength. The densely selected 1100–1200 nm interval (including 1102.4 nm, 1113.6 nm, 1158.2 nm, and 1186.0 nm) corresponds to the second overtone of C–H bonds, commonly linked to sugar-related absorption. Wavebands such as 1402.7 nm fall directly within the O–H first overtone region, which is highly sensitive to internal moisture redistribution and water exudation caused by tissue rupture. Furthermore, features around 1756.8 nm (along with 1773.3 nm and 1778.8 nm) reside in the first overtone region of C–H bonds, reflecting metabolic changes in organic acids and the cell–matrix. Finally, the bands in the longer wavelength region, such as 2131.9 nm, 2181.5 nm, and 2485.0 nm, are located in combination absorption regions consistently associated with pectin and structural polysaccharide degradation [67].

Crucially, these chemically meaningful spectral regions are highly consistent with the high-frequency zones identified in the stability analysis, suggesting that the statistically important wavebands may be associated with under-lying tissue alterations. Such statistical–physical consistency enhances the interpretability of the selected feature subset. From a measurement perspective, the alignment between model-derived importance and known absorption mechanisms supports the rationality of dimensionality reduction and provides guidance for potential multispectral system design.

#### 3.2.5. Comparative Analysis of Different Waveband Selection Results

**Figure 10:**
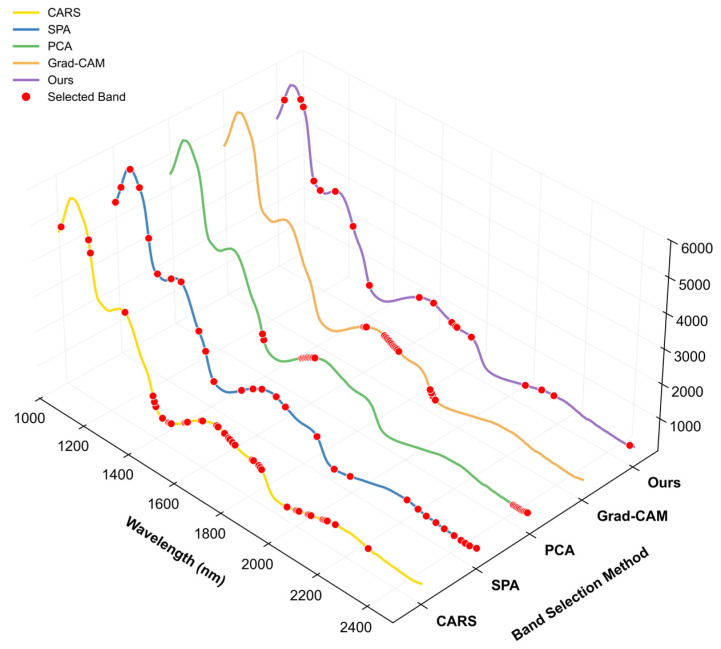
Comparative diagram of waveband selection results by different methods.

To evaluate the effectiveness of SR-IGWO, its performance was compared with four commonly used waveband selection methods, including PCA, SPA, CARS, and Grad-CAM derived from a 1D-CNN model. To ensure a strictly fair and objective comparison, all baseline methods were carefully configured and evaluated under identical conditions using the exact same isolated test dataset and the same RF classifier. Specifically, PCA evaluated waveband importance by weighting absolute projection loadings with explained variance ratios, followed by a PLS forward search to identify the subset minimizing the Root Mean Square Error; CARS was executed with 50 Monte Carlo sampling runs and 10-fold cross-validation to select informative variables; SPA employed successive orthogonal vector projections to minimize collinearity among adjacent spectral channels, determining the final variable count via Multiple Linear Regression (MLR); and Grad-CAM was derived from a custom 1D-CNN backbone (Conv1D layers + adaptive average pooling), extracting highly weighted discrete wavebands by computing normalized global average pooled gradients multiplied by the final convolutional activation maps. As shown in Figure 10, the number and distribution of selected wavebands varied among methods. PCA, SPA, CARS, and Grad-CAM selected 24, 29, 41, and 20 wavebands, respectively, while SR-IGWO retained only 18 wavebands. PCA and CARS tended to select clustered bands within certain spectral regions, whereas SPA produced a relatively scattered but larger subset. Grad-CAM mainly focused on narrow spectral intervals. In contrast, SR-IGWO selected more evenly distributed wavebands across the spectrum, which helped reduce redundancy while preserving complementary spectral information.

**Figure 11:**
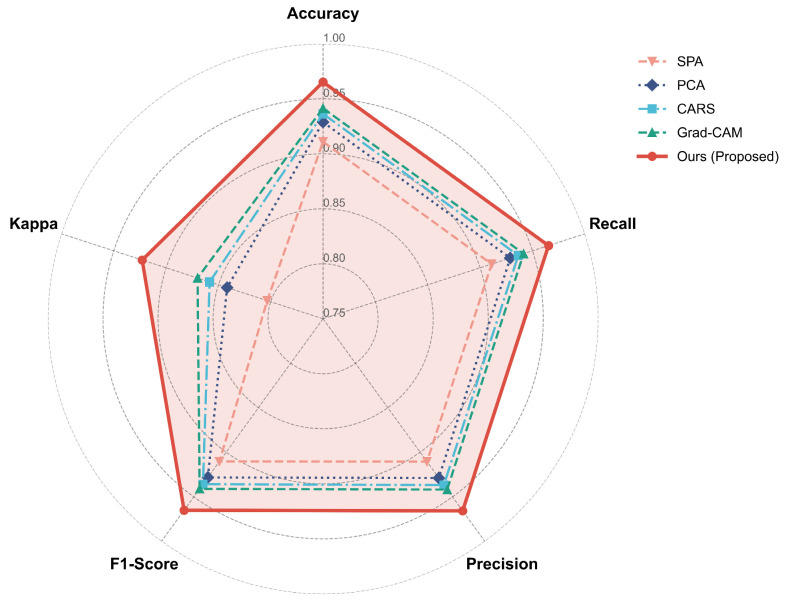
Radar chart of performance metrics for different waveband selection methods.

The classification performance obtained from different waveband subsets is summarized in Table 2 and visualized in Figure 11. Although methods such as CARS and PCA retained more wavebands, their classification performance did not show corresponding improvement. SPA exhibited the lowest performance across most evaluation metrics. Grad-CAM achieved competitive results but showed slightly lower Kappa values. The SR-IGWO subset achieved the highest observed performance with an accuracy of 0.9653 and a Kappa coefficient of 0.9229 while using the smallest number of wavebands. These results indicate that SR-IGWO can effectively extract compact and informative spectral features for early apple bruise detection.

### 3.3. Analysis of Bruise Identification Results

#### 3.3.1. Comparative Evaluation with Different Classification Models

**Figure 12:**
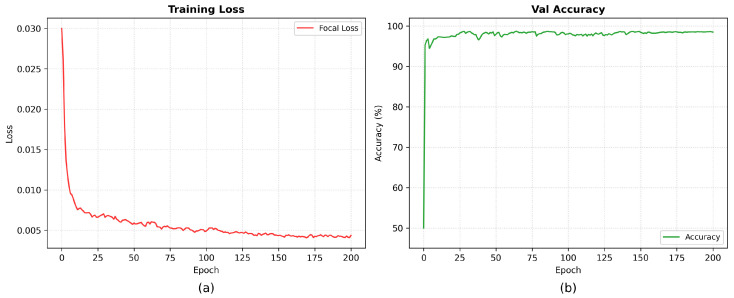
Convergence curves during the training of the CNN-Transformer model: (**a**) Training loss; (**b**) Validation accuracy.

Based on the 18 discrete wavebands selected by SR-IGWO, the DSFormer classification model was constructed for early bruise detection. To reduce potential overfitting under low-dimensional input and enhance the detection of hard-to-classify samples, Focal Loss was adopted during training to prevent excessive parameter updates after convergence. The training dynamics over 200 epochs are shown in Figure 12 (representative run with random seed = 42). As illustrated in Figure 12a, the training loss decreases rapidly during the initial stage and then gradually stabilizes with minor oscillations. Figure 12b shows the evolution of validation accuracy. After an initial fluctuation stage, the accuracy gradually increases and stabilizes above 98.5% after approximately 150 epochs, indicating stable convergence behavior under the selected discrete waveband configuration. These results suggest that the proposed architecture can achieve consistent and stable training behavior under the selected discrete waveband configuration.

To further evaluate classification performance, DSFormer was compared with five representative machine learning and deep learning models, including SVM, Long Short-Term Memory (LSTM) [68], RF [69], 1D-CNN, and PLS-DA. To assess optimization consistency under different random initializations, all models were repeatedly trained and evaluated over 10 runs using different random seeds (42, 1024, 2026, 888, 999, 123, 456, 789, 2025, and 314), while sharing the same feature subset and data partition strategy. The results are reported as Mean ± Standard Deviation, supplemented by 95% confidence intervals (CI) estimated from repeated runs (Table 3). All models achieved relatively high overall accuracy (>96%), suggesting that the selected wavebands retain meaningful discriminative information under the current controlled dataset and experimental conditions for bruise detection. Notably, traditional machine learning models (e.g., SVM and PLS-DA) produced identical results across repeated runs due to their deterministic optimization behavior under fixed data partitions, whereas deep learning models reflected stochastic variations associated with random initialization. As summarized in Table 3, DSFormer achieved the highest observed performance among the evaluated models across all evaluation metrics, reaching an Accuracy of 0.9911 ± 0.0008 (95% CI: [0.9905, 0.9917]), a Damage Precision of 0.9587 ± 0.0082 (95% CI: [0.9528, 0.9646]), a Damage Recall of 0.9604 ± 0.0108 (95% CI: [0.9527, 0.9682]), and a Damage F1-score of 0.9595 ± 0.0039 (95% CI: [0.9567, 0.9622]). In contrast, several baseline models (e.g., PLS-DA and RF) achieved relatively higher Precision but substantially lower Recall, indicating a tendency to miss subtle bruise samples. Such missed detections are undesirable in practical early bruise inspection scenarios. In addition, LSTM exhibited relatively larger variability across repeated runs, suggesting reduced stability under sparse discrete spectral inputs.

To further examine whether the observed performance improvements were consistently maintained across repeated runs, non-parametric statistical comparisons were conducted on the F1-scores [70]. Specifically, paired comparisons between DSFormer and other model were performed using the Wilcoxon signed-rank test, and Holm–Bonferroni correction was applied to control for multiple comparisons (Table 4). In addition, mean performance differences together with their corresponding 95% confidence intervals were calculated to quantify the magnitude of the observed improv ements. The statistical comparisons indicate that DSFormer consistently achieved higher F1-scores than all baseline models under the repeated experimental conditions on the current dataset (adjusted *p* < 0.01). In particular, DSFormer improved the F1-score by 5.28% compared with 1D-CNN and by 12.56% compared with PLS-DA. These findings provide statistical support for the observed comparative performance trends under repeated optimization runs, while emphasizing that they reflect model stability on the current dataset rather than generalized population-level robustness.

To complement the averaged statistical results presented in Table 4, one representative run (random seed = 42) was selected for subsequent qualitative visualization and pixel-level analysis. The performance obtained in this representative run remained consistent with the overall multi-run statistical trends. Compared with conventional baseline models (e.g., PLS-DA and RF), DSFormer maintained a more balanced trade-off between Recall (0.9596), Precision (0.9640), and F1-score (0.9622), particularly under sparse discrete spectral inputs. In contrast, several baseline methods exhibited relatively higher Precision but noticeably lower Recall, suggesting reduced sensitivity to subtle early bruising signals. These observations are consistent with the repeated-run statistical analyses and further support the observed comparative advantage of the proposed architecture under the current dataset for low-dimensional hyperspectral bruise detection.

#### 3.3.2. Model Sensitivity Analysis

To directly evaluate the sensitivity of the selected 18 discrete wavebands to weak bruising signals, the predictions from the final DSFormer model were further stratified according to the predefined impact levels (10 cm, 15 cm, and 25 cm) in the independent test set [71], and performance metrics were calculated separately over ten repeated runs. The quantitative results are summarized in Table 5.

As impact severity decreases, the physiological and structural changes induced in subepidermal tissues become increasingly subtle, making them more difficult to distinguish from natural biological variability. Consistent with this expectation, a gradual decline in detection performance was observed from severe to mild bruising conditions. Specifically, for the 10 cm impact group—representing the weakest bruising condition in this study—the model still achieved a recall of 92.03% and an F1-score of 93.96%, indicating effective sensitivity to subtle bruise-related spectral variations within the evaluated dataset. For the 15 cm and 25 cm groups, recall further increased to 97.04% and 99.05%, respectively, accompanied by progressively lower standard deviations across repeated runs. This reduction in variance suggests improved prediction consistency under stronger bruise-related spectral responses. Overall, these results suggest that the selected 18 discrete wavebands, when combined with the DSFormer architecture, retain discriminative sensitivity to weak spectral perturbations associated with early-stage mechanical bruising within the current dataset and controlled experimental conditions.

### 3.4. Comparative Analysis of Apple Bruise Visualization

To evaluate localization accuracy and boundary reconstruction capability, a pixel-level visualization analysis was conducted based on model predictions over the entire apple surface. As described in Section 2.7.1, all visualizations were performed on strictly unseen test samples, supporting a fruit-wise isolated evaluation of spatial prediction consistency under the current dataset. The spatial prediction maps were generated through a reconstruction-based inference process. First, the hyperspectral cube was reshaped into a two-dimensional matrix (pixels × spectral bands) [72]. The spectral dimension was then reduced to 18 bands using the SR-IGWO selection mask. Each pixel vector was independently fed into the trained model to produce a bruise probability. The predicted probabilities and binary labels were subsequently remapped to their original spatial coordinates to reconstruct two-dimensional outputs. This process yields both continuous probability heatmaps and binary prediction maps for bruise localization.

#### 3.4.1. Quantitative Spatial Evaluation

Two standard spatial evaluation metrics from image segmentation—IoU and the Dice Coefficient—were introduced to quantitatively assess the agreement between predicted binary maps and manual ground truth masks. The quantitative results, derived from the representative run (random seed = 42), are summarized in Table 6. The proposed method achieved the highest spatial agreement, with an IoU of 0.9290 and a Dice coefficient of 0.9632. In comparison, baseline models exhibited lower spatial consistency. SVM achieved the second-best performance (IoU: 0.8088, Dice: 0.8943), while LSTM and 1D-CNN showed reduced boundary integrity. PLS-DA yielded the lowest spatial agreement. These results suggest that the proposed framework provides higher observed spatial agreement and more consistent boundary reconstruction under reduced spectral dimensionality.

#### 3.4.2. Qualitative Visual Comparison

**Figure 13:**
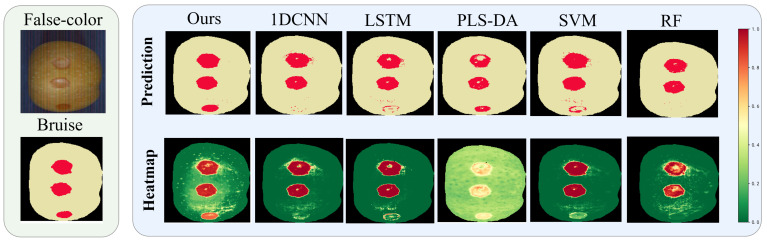
Comparison of bruise visualization results produced by different models.

The quantitative findings are further supported by the qualitative visual results. Figure 13 shows the visualization results of the proposed model and five comparative models (1D-CNN, LSTM, RF, PLS-DA, and SVM) on representative bruised samples, generated using the aforementioned representative models. The first column presents the original false-color images and corresponding ground truth masks. The remaining columns show the binary prediction maps (Prediction, red indicates bruise regions) and bruise probability heatmaps (Heatmap, color gradient from green to red represents increasing bruise confidence). As illustrated in Figure 13, noticeable differences are observed in bruise boundary integrity and the detection of small-scale bruise regions. The 1D-CNN, LSTM and RF models exhibit irregular edge noise and incomplete identification of minor bruises, resulting in missed detections in some regions (reflecting their lower IoU scores around 0.75–0.77). PLS-DA and SVM provide relatively stable contour extraction for major bruise areas; however, their heatmaps show higher confidence fluctuations in healthy regions, which may lead to false positives under uneven illumination or natural peel variations.

In contrast, the proposed approach produces prediction maps that closely match the annotated bruise regions. The reconstructed bruise areas show more continuous boundaries with fewer isolated noise pixels. Moreover, the probability heatmaps exhibit smoother confidence transitions around bruise regions, indicating more consistent spatial responses. These results suggest that the proposed framework showed consistent bruise localization and boundary reconstruction under reduced spectral dimensionality, suggesting its potential utility for hyperspectral bruise inspection under controlled experimental conditions.

#### 3.4.3. Cross-Cultivar External Validation

To preliminarily evaluate the cross-cultivar transferability of the proposed framework, a preliminary zero-shot external validation was conducted using an independently acquired dataset of Luochuan apples (a different cultivar from the original Fuji) [73]. As fruit optical properties vary significantly across cultivars due to differences in peel thickness, background coloration, and tissue structure, this test provides a preliminary evaluation of model behavior under cultivar-induced domain shifts. All models, which were pre-trained solely on the original Fuji apple dataset (representative run with random seed = 42), were directly applied to this unseen external dataset without any fine-tuning or re-calibration. The comparative spatial metrics are summarized in Table 7.

As expected, inter-cultivar variations caused a performance drop across all models compared to intra-cultivar results. Conventional baselines (PLS-DA, RF) suffered severe spatial degradation (IoU dropping to 0.1663 and 0.4005, respectively), suggesting stronger dependence on the primary cultivar’s spectral background. In contrast, the proposed DSFormer maintained relatively consistent spatial localization on the evaluated supplementary cultivar under the current controlled experimental setting (overall accuracy ~95.8%, IoU = 0.7686, Dice = 0.8721).

**Figure 14:**
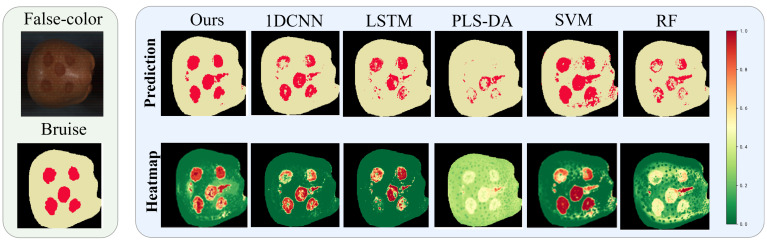
Qualitative visualization of cross-cultivar transfer performance for early bruise detection across different models.

These quantitative findings are corroborated by the qualitative visual comparisons in Figure 14. Under cultivar-induced domain shift, baseline models showed obvious degradation: PLS-DA largely failed to detect bruises (low-confidence heatmaps); RF produced fragmented predictions with high background noise; SVM gave false positives along fruit boundaries; deep learning baselines (1D-CNN, LSTM) exhibited irregular edge noise and fragmented pixel predictions. By contrast, DSFormer preserved accurate boundary reconstruction with minimal background interference, closely matching the ground truth. This demonstrates that the selected wavebands (via SR-IGWO) and the Transformer’s global dependency modeling may capture bruise-related spectral characteristics that are less dependent on cultivar-specific backgrounds.

These preliminary results suggest that the proposed framework may preserve useful discriminative capability under limited cultivar variation. However, we acknowledge that this validation is limited to a single supplementary cultivar under controlled laboratory conditions; thus, extensive multi-cultivar and multi-batch evaluations in dynamic handling environments are still required before generalizing to real-world postharvest deployments.

### 3.5. Ablation Experiments

To quantitatively evaluate the contribution of each functional component within the proposed DSFormer architecture, a structured ablation study was conducted. Four modified configurations were designed by isolating key modules: (1) Variant A (Local CNN Embedding), replacing the pointwise embedding with a conventional 5 × 5 1D convolution; (2) Variant B (No Transformer), removing the Transformer encoder to evaluate the role of global dependency modeling; (3) Variant C (No Positional Encoding), excluding positional encoding; and (4) Variant D (Cross Entropy Loss), replacing Focal Loss with standard Cross-Entropy Loss to assess its influence on hard-to-classify samples [74]. To ensure fair comparison, all ablation variants were trained and evaluated under the same data partition, feature subset, and model-selection protocol as the full model. In addition, each configuration was independently executed over 10 runs using different random seeds to reduce the influence of stochastic training variability. The quantitative results are summarized in Table 8 as Mean ± Standard Deviation (SD), together with the absolute performance changes (ΔF1 and ΔRecall) relative to the full model.

Among all variants, removing the Transformer encoder (Variant B) caused the largest performance degradation, reducing the average F1-score by 2.90% and Recall by 2.38% relative to the full model. This result indicates that global dependency modeling plays a critical role in capturing cross-band relationships under sparse discrete spectral inputs. In addition, replacing the pointwise embedding with conventional local convolution (Variant A) or excluding positional encoding (Variant C) also resulted in noticeable performance reductions. These observations suggest that imposing local continuity constraints on physically discrete wavebands may introduce redundant feature coupling, while relative spectral positioning remains beneficial for representing sparse spectral signatures. An interesting trade-off was observed in the loss function ablation (Variant D). Although replacing Focal Loss with standard Cross-Entropy Loss produced a nearly unchanged F1-score, the Recall decreased by 0.79%. This finding indicates that Focal Loss appears to improve recall sensitivity under the current dataset for subtle bruise samples, which is desirable for early bruise inspection tasks where missed detections are more critical than occasional false positives. Overall, the ablation results quantitatively and statistically support the complementary roles of discrete pointwise embedding, global self-attention modeling, and recall-oriented optimization in early bruise detection under sparse spectral conditions, while acknowledging that these findings are specific to the current experimental dataset and controlled conditions.

## 4. Discussion

In this study, the SR-IGWO algorithm identified an 18-waveband subset from continuous hyperspectral data to detect early apple bruising. Previous hyperspectral bruise-detection studies have primarily focused either on full-spectrum deep learning frameworks or on conventional waveband-selection methods combined with shallow classifiers [14,36], while relatively limited attention has been paid to the interpretability of sparse discrete spectral modeling. In this work, SHAP-based analysis was incorporated to provide additional insight into the spectral-band-level contributions associated with the detection process. Based on SHAP analysis, highly weighted wavebands such as 1839.6 nm and 1402.7 nm correspond to known absorption regions associated with water (O–H) and structural carbohydrates (C–H/C–O). This observation suggests that early bruising may involve sub-epidermal moisture redistribution and localized cell wall degradation. However, these interpretations should be considered with caution. The SHAP analysis only explains feature importance within the Random Forest model used during the band-selection stage, rather than the DSFormer classifier itself [75]. This methodological distinction means the inferred biochemical associations, while consistent with existing literature, remain strictly correlative rather than causative, and require further validation through direct biochemical assays to establish a definitive link [76,77].

At the modeling level, conventional sequence-based approaches rely on local spectral continuity, which may be suboptimal when inputs consist of sparsely distributed discrete wavebands [68]. Therefore, the present work does not aim to replace existing full-spectrum hyperspectral learning paradigms but rather to explore whether structurally matched modeling strategies can improve representation efficiency under heavily compressed discrete spectral conditions. The DSFormer architecture attempts to address this issue by embedding discrete wavebands and modeling their global relationships using a self-attention mechanism. Beyond architectural considerations, we also incorporated repeated-run statistical analyses to assess performance reproducibility and uncertainty. Specifically, all models were independently evaluated over ten runs using different random seeds, and the resulting metrics were further analyzed using confidence intervals and non-parametric statistical comparisons [78]. Although these analyses improve the reliability of the reported performance trends under the current experimental setting, statistical reproducibility observed on a controlled dataset does not inherently guarantee equivalent robustness under unseen cultivars, acquisition systems, or industrial processing environments. Nevertheless, the relatively low variance observed across repeated runs suggests that the proposed framework maintains stable optimization behavior under sparse spectral inputs. This design helps the network concentrate on subtle spectral variations associated with early bruising while reducing the risk of missed detections [79].

A key methodological concern in pixel-level hyperspectral analysis is the potential dependence among samples, particularly due to spatial correlation and ROI overlap within the same fruit. To mitigate this issue, the dataset was constructed from 100 independent apples, yielding over 11,000 spectral samples (approximately 5700 bruised and 6000 healthy regions). A strict fruit-wise partitioning strategy was adopted to ensure that all samples from the same apple were assigned exclusively to a single subset, thereby substantially helping reduce the risk of direct data leakage between training and testing phases. Nevertheless, because multiple pixel-level ROIs extracted from the same fruit inherently share similar physiological and structural characteristics, residual intra-fruit correlations may still persist. Consequently, the reported performance should be interpreted as reflecting reproducible discrimination capability under the current controlled experimental setting, rather than fully independent population-level generalization.

Despite the promising results obtained in this study, several methodological limitations should be explicitly acknowledged when considering the broader applicability of the proposed framework [70]. The current experiments were conducted using a single apple cultivar under controlled impact conditions and within a relatively narrow post-impact time window (1–4 h). Consequently, the spectral distributions represented in this study may not fully capture the variability encountered in practical postharvest environments, where cultivar-dependent optical properties, illumination conditions, surface characteristics, storage states, and handling procedures may differ substantially [15,63]. Although a preliminary cross-cultivar external validation was incorporated in the study, the additional evaluation was still conducted under controlled laboratory conditions using only one supplementary cultivar. Therefore, the current validation should not yet be regarded as comprehensive external validation across diverse industrial scenarios or large-scale postharvest processing environments. Furthermore, although repeated-run statistical analyses improved the reliability assessment of the reported performance, the overall experimental diversity remains relatively limited. Under practical distribution shifts, performance consistency across broader application scenarios remains to be further verified. Accordingly, the proposed framework should presently be regarded as a preliminary but encouraging step toward efficient low-dimensional hyperspectral bruise detection, while further large-scale multi-cultivar, multi-device, and multi-environment validation remains necessary before practical deployment.

## 5. Conclusions

This study developed a hyperspectral framework integrating waveband optimization and discrete spectral modeling for early apple bruise detection. Using the SR-IGWO algorithm, 18 representative wavebands were selected from 273 original spectral channels, achieving a 93.4% reduction in spectral dimensionality. Stability analysis across 20 repeated optimization runs indicated that the selected bands consistently concentrated on spectrally meaningful regions under the current experimental setting.

The proposed DSFormer model, specifically designed for discrete waveband inputs, achieved a classification accuracy of 99.11% ± 0.08%, a recall of 96.04% ± 1.08%, and an F1-score of 95.95% ± 0.39% on the independent test set. Repeated-run statistical analyses further suggested that the observed performance improvements remained relatively stable under different random initializations within the current dataset. Compared with conventional methods (e.g., SVM, PLS-DA, and 1D-CNN), DSFormer achieved improved recall and F1-score under substantially reduced spectral dimensionality. Ablation experiments further suggested that pointwise embedding, Transformer-based global dependency modeling, and Focal Loss collectively contributed to improved bruise detection performance under sparse spectral input conditions. Visualization results additionally demonstrated spatially coherent bruise localization with relatively limited background interference under controlled imaging conditions.

Nevertheless, several important limitations should be acknowledged when interpreting these findings. The current study was conducted using a single apple cultivar under controlled laboratory conditions and within a limited post-impact time window. Although fruit-wise partitioning, repeated-run statistical analyses, and preliminary cross-cultivar evaluation were incorporated to improve evaluation reliability, the framework has not yet undergone extensive large-scale external validation across diverse cultivars, acquisition batches, imaging systems, or practical industrial processing environments. Consequently, the reported robustness and generalization capability should presently be interpreted within the scope of the current experimental setting rather than as evidence of fully established real-world applicability.

Overall, the present study provides a preliminary but encouraging exploration of combining targeted waveband selection with structurally matched discrete spectral modeling for efficient early bruise detection. Further multi-domain validation and real-world evaluation remain necessary before broader practical deployment can be considered.

## Figures and Tables

**Table 1:** Biochemical mechanisms and vibrational assignments of optimized feature bands.

Band Range (nm)	Vibration Mode	Biochemical Component/Physiological Significance
1100–1300	C-H 2nd Overtone	Sugars (Changes in glucose/fructose content)
1350–1450	O-H 1st Overtone	Free water (Cell rupture, water exudation)
1600–1800	C-H 1st Overtone	Organic acids, Cellulose (Cell–matrix metabolism)
1800–1950	O-H + C-O Comb.	Cellulose, Bound water (Structural disintegration)
2100–2500	C-H + C-C Comb.	Cellulose, Pectin (Cell wall structural disintegration)

**Table 2:** Comparative performance of different waveband selection strategies.

Method	Accuracy	Recall	Precision	F1-Score	Kappa
Grad-CAM	0.9415	0.9415	0.9418	0.9410	0.8700
CARS	0.9364	0.9364	0.9367	0.9358	0.8585
PCA	0.9288	0.9288	0.9288	0.9282	0.8418
SPA	0.9110	0.9110	0.9106	0.9105	0.8033
Ours	0.9653	0.9653	0.9657	0.9650	0.9229

**Table 3:** Performance comparison of classification models over 10 repeated runs (Mean ± SD [95% CI]).

Method	Ours	SVM	LSTM	RF	1D-CNN	PLS-DA
Accuracy	0.9911 ± 0.0008 [0.9905, 0.9917]	0.9772	0.9787 ± 0.0066 [0.9740, 0.9834]	0.9734 ± 0.0002 [0.9733, 0.9736]	0.9805 ± 0.0043 [0.9775, 0.9836]	0.9688
Recall	0.9604 ± 0.0108 [0.9527, 0.9682]	0.8833	0.8628 ± 0.0636 [0.8173, 0.9084]	0.7844 ± 0.0019 [0.7831, 0.7858]	0.8707 ± 0.0448 [0.8386, 0.9028]	0.7155
Precision	0.9587 ± 0.0082 [0.9528, 0.9646]	0.9056	0.9376 ± 0.0100 [0.9305, 0.9448]	0.9662 ± 0.0019 [0.9648, 0.9675]	0.9471 ± 0.0054 [0.9433, 0.9510]	0.9992
F1-Score	0.9595 ± 0.0039 [0.9567, 0.9622]	0.8943	0.8976 ± 0.0346 [0.8728, 0.9223]	0.8659 ± 0.0009 [0.8652, 0.8665]	0.9067 ± 0.0227 [0.8904, 0.9230]	0.8339

Note: Results are reported as Mean ± SD with 95% confidence intervals (CI). Confidence intervals are omitted for deterministic models that produced identical results across repeated runs.

**Table 4:** Pairwise statistical comparisons between DSFormer and baseline models (F1-score).

Comparison	Mean Difference [95% CI]	Adjusted *p*-Value (Holm)
DSFormer vs. SVM	0.0652 [0.0624, 0.0679]	0.0098
DSFormer vs. LSTM	0.0619 [0.0381, 0.0857]	0.0098
DSFormer vs. RF	0.0936 [0.0906, 0.0966]	0.0098
DSFormer vs. 1D-CNN	0.0528 [0.0359, 0.0696]	0.0098
DSFormer vs. PLS-DA	0.1256 [0.1228, 0.1284]	0.0098

**Table 5:** Breakdown of DSFormer classification performance by impact level (Mean ± SD).

Impact Level	Accuracy	Recall (Bruise)	F1-Score
10 cm (Mild)	0.9853 ± 0.0015	0.9203 ± 0.0152	0.9396 ± 0.0061
15 cm (Moderate)	0.9921 ± 0.0007	0.9704 ± 0.0084	0.9620 ± 0.0032
25 cm (Severe)	0.9959 ± 0.0002	0.9905 ± 0.0035	0.9769 ± 0.0015

**Table 6:** Quantitative spatial evaluation of bruise localization performance.

Method	Ours	SVM	LSTM	RF	1D-CNN	PLS-DA
Accuracy	0.9916	0.9772	0.9734	0.9732	0.9714	0.9688
IoU	0.9290	0.8088	0.7713	0.7610	0.7546	0.7150
Dice Coefficient	0.9632	0.8943	0.8709	0.8643	0.8601	0.8339

**Table 7:** Quantitative spatial evaluation on the external cross-cultivar dataset.

Method	Ours	SVM	LSTM	RF	1D-CNN	PLS-DA
Accuracy	0.9583	0.9267	0.9203	0.8980	0.9423	0.8626
IoU	0.7686	0.6705	0.5228	0.4005	0.6537	0.1663
Dice Coefficient	0.8721	0.8028	0.6866	0.5719	0.7906	0.2852

**Table 8:** Quantitative ablation study results over 10 independent runs.

Model	Damage F1 (Mean ± SD)	ΔF1	Damage Recall (Mean ± SD)	ΔRecall
Full_Model	0.9595 ± 0.0039	-	0.9604 ± 0.0108	-
Variant A	0.9555 ± 0.0043	−0.0040	0.9575 ± 0.0102	−0.0029
Variant B	0.9305 ± 0.0103	−0.0290	0.9366 ± 0.0227	−0.0238
Variant C	0.9515 ± 0.0068	−0.0080	0.9465 ± 0.0091	−0.0139
Variant D	0.9597 ± 0.0027	+0.0002	0.9525 ± 0.0050	−0.0079

Note: ΔF1 and ΔRecall represent the absolute difference in mean scores compared to the Full Model.

## Data Availability

The raw data supporting the conclusions of this article will be made available by the authors on request.
